# mTOR inhibitors in the pharmacologic management of tuberous sclerosis complex and their potential role in other rare neurodevelopmental disorders

**DOI:** 10.1186/s13023-017-0596-2

**Published:** 2017-03-14

**Authors:** David N. Franz, Jamie K. Capal

**Affiliations:** 1 Department of Pediatrics, Tuberous Sclerosis Clinic, Cincinnati Children’s Hospital Medical Center, University of Cincinnati College of Medicine, Cincinnati, OH USA; 2 Department of Neurology, Tuberous Sclerosis Clinic, Cincinnati Children’s Hospital Medical Center, University of Cincinnati College of Medicine, Cincinnati, OH USA

**Keywords:** Hamartomas, Morbidity, Mammalian target of rapamycin inhibitors, Neurologic manifestations, Tuberous sclerosis complex

## Abstract

Tuberous sclerosis complex (TSC) is a rare autosomal dominant genetic disorder that affects multiple organ systems throughout the body. Dysregulation of the mammalian target of rapamycin (mTOR) pathway is implicated in the disease pathology, and evidence exists to support the use of mTOR inhibitors in treatment. The mTOR pathway has also been investigated as a potential treatment target for several other rare diseases. TSC research has highlighted the value of pursuing targeted therapies based on underlying molecular pathophysiology. One goal of current research is to identify the role of mTOR inhibition in neurologic and developmental disorders apart from TSC. There is also particular interest in the potential role of mTOR inhibitors in preventing seizures, neurodevelopmental disabilities, renal tumors, cutaneous tumors, and other manifestations typically seen in TSC. It is foreseeable that use of mTOR inhibition to prevent long-term morbidity in TSC will become mainstream therapeutic practice. This review will provide an overview of the relationship between the mTOR pathway and TSC disease pathology, summarize the clinical evidence supporting the use of mTOR inhibitors for treatment of the various manifestations of TSC, and discuss the potential therapeutic role of mTOR inhibitors in several rare diseases.

## Background

Tuberous sclerosis complex (TSC) is an autosomal dominant genetic disorder that typically results in the growth of hamartomas in multiple major organ systems [1–3]. TSC is a rare disease that is estimated to occur in one in 6000 births, affecting approximately 1.5 million people worldwide [1, 2, 4]. Although TSC can manifest in many organs to different degrees of severity, it is primarily characterized by neurologic manifestations (including seizures); neurodevelopmental disabilities (e.g., intellectual disability and autism); and lesions in the brain, lungs, kidneys, and skin [1]. Updated TSC consensus recommendations established in 2012 now recommend the use of systemic treatment with mammalian target of rapamycin (mTOR) inhibitors in certain cases, which provides an opportunity to treat multiple manifestations of TSC simultaneously [5]. This review evaluates the current role of and available clinical data on mTOR inhibitor use in TSC and discusses potential future roles for mTOR inhibitors in TSC and similar diseases that are currently under investigation.

## Materials and methods

We conducted a search of the published literature on PubMed/Medline for, and participated in, key clinical studies of mTOR inhibitors in treating subependymal giant cell astrocytomas (SEGAs), angiomyolipomas, lymphangioleiomyomatosis (LAM), angiofibromas, and epilepsy in patients with TSC. For other rare diseases, the terms “mTOR”, “mTOR inhibitor”, and “mTOR inhibition” were used in individual searches for “Leigh syndrome”, “Down syndrome”, and “Neurofibromatosis”.

## Manifestations associated with TSC

Cortical tubers, subependymal nodules (SENs), and SEGAs are the primary abnormalities found in the brain [1]. Cortical tubers, which are formed during embryogenesis, can be present at birth and have been observed in 80% to 90% of patients with TSC [1, 2, 6]. Tubers are thought to be associated with the development of seizures, intellectual disability, behavioral difficulties, and autism [6, 7]. SENs, which are asymptomatic hamartomas that protrude into the ventricles of the brain, occur in approximately 90% of individuals, and in about 5% to 20% of individuals they can develop into SEGAs [1, 8]. SEGAs are slow-growing glioneuronal tumors that develop near the foramen of Monro and have the potential to cause hydrocephalus, increased intracranial pressure, and death secondary to impeded ventricular cerebrospinal fluid flow [1, 6]. Prior to the use of pharmacologic therapy to reduce tumor volume, treatment for growing, symptomatic SEGAs has been mainly surgical resection [9, 10].

In the central nervous system, epilepsy is the most common medical disorder in patients with TSC, affecting up to 96% of individuals [11, 12]. Focal seizures and infantile spasms are the most common seizure types in patients with TSC. Infantile spasms are common during infancy (i.e., first year of life), occurring in up to one-third of children with TSC [10, 11]. Early onset of infantile spasms is associated with poor developmental outcomes and worse future seizure control [1, 13, 14].

TSC involves multiple organ systems, including the brain, kidneys, lungs, heart, and skin. Renal manifestations occur in approximately 55% to 90% of patients with TSC, with angiomyolipomas occurring in up to 75% of patients [6]. Renal angiomyolipomas are the most common cause of mortality in patients with TSC because of potential renal failure or hemorrhage [15]. LAM is the most common lung manifestation in TSC, characterized by development of cystic lesions in the lung. LAM can occur in patients with TSC but can also occur sporadically in non-TSC individuals [16]. LAM occurs in approximately 30% to 40% of patients with TSC, is seen almost exclusively in women, and can lead to destruction of lung parenchyma, resulting in progressive dyspnea on exertion and recurrent pneumothorax [16–18]. Cardiac rhabdomyomas are a common initial manifestation of TSC and occur in 33% of patients. Rhabdomyomas are typically asymptomatic and spontaneously regress with age [19, 20]; however, in rare instances, the location of the tumor can cause arrhythmia and heart failure [21]. Most patients (> 90%) exhibit skin manifestations, including hypomelanotic macules (ash leaf spots), angiofibromas and/or cephalic plaques, ungual or periungual fibromas, shagreen patches, and confetti skin lesions. Angiofibromas are present in approximately 80% of individuals with TSC older than the age of 5 years, and typically develop on the face [3]. The characteristic red or pink nodules often develop within the first few years of life and become more pronounced with age [22]. Although multiple dermatologic treatments exist (including surgical excision in some cases), fibroma often recurs [22].

## mTOR pathway in the pathogenesis of TSC

**Fig. 1:**
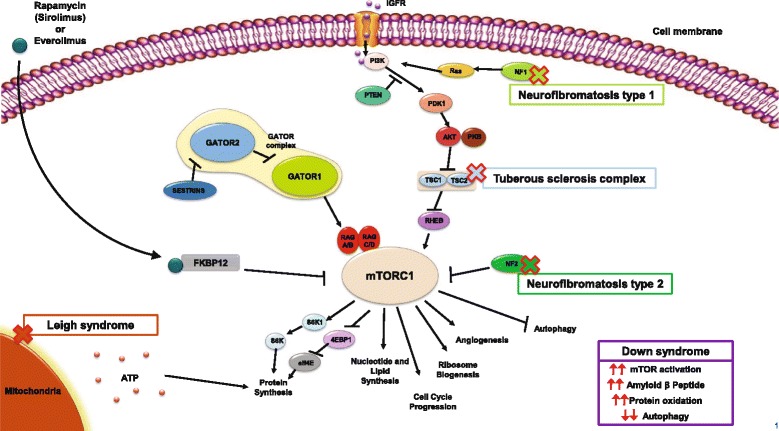
The mammalian target of rapamycin (mTOR) signaling pathway and possible involvement of rare diseases in the pathway. Stimulants such as insulin-like growth factor bind to tyrosine kinase receptors, which leads to the phosphorylation of phosphatidylinositol 3-kinase (*PI3K*) [23]. A cascade of subsequent phosphorylation events results in the activation of protein kinase B (AKT), which in turn phosphorylates and inhibits the TSC1/TSC2 complex, a negative regulator of mTOR that is directed against the positive regulator Ras homolog enriched in brain (Rheb). As a result, inhibition of the TSC1/TSC2 complex results in the activation of mTOR [23–25]. *NF1*-encoded neurofibromin and *NF2*-encoded Merlin proteins also act as negative regulators of the mTOR pathway. Neurofibromin functions as a Ras-GTPase activating protein that inhibits the actions of Ras on PI3K [26], while Merlin acts directly on mTOR complex 1 (mTORC1) [27]. Sirolimus and everolimus both bind to and form complexes with FK506-binding protein-12 (FKBP12), resulting in the inhibition of mTORC1 activity [24]. While mechanisms are complex and not fully clear in Leigh and Down syndrome, evidence has shown a relationship between mTOR activity and ATP (*Leigh syndrome*), and decreased autophagy with increased protein production and oxidation with mTOR hyperactivation (*Down syndrome*) [62, 65, 66]

In normal cells, the mTOR signaling cascade (also known as the phosphatidylinositol 3-kinase [PI3K]/protein kinase B [Akt]/mTOR pathway) plays an important role in cell growth, proliferation, and survival (Fig. 1) [23]. Stimulants, such as growth factors (e.g., insulin-like growth factor-1 [IGF-1]), bind to tyrosine kinase receptors (e.g., IGF-1R), which leads to the phosphorylation of PI3K [23]. This activation of PI3K results in a cascade of phosphorylation events, resulting in the activation of Akt, which in turn inhibits the TSC1/TSC2 complex, which negatively regulates mTOR by acting as a GTPase-activating protein toward Ras homolog enriched in brain (Rheb), a direct and positive regulator of mTOR. As a result, inhibition of the TSC1/TSC2 complex results in the overactivation of mTOR, leading to cell growth and proliferation [23–25]. Two additional proteins, the *NF1*-encoded neurofibromin and the *NF2*-encoded Merlin, also act as negative regulators of the mTOR pathway [26, 27]. mTOR forms two distinct multiprotein complexes, mTORC1 and mTORC2, which are differentiated by their interaction partners (regulatory associated protein of mTOR [RAPTOR] for mTORC1 and rapamycin-insensitive companion of mTOR [RICTOR]/SIN1 for mTORC2), substrate selectivity, and sensitivity to rapamycin (sirolimus) and its analogs (e.g., everolimus) [25, 28]. The downstream effects of mTORC1 include gene transcription and protein translation, cell proliferation and survival, and angiogenesis, while mTORC2 is thought to mediate cytoskeletal dynamics [28]. Dysregulation of the mTOR pathway has been implicated in the development of many cancers, including TSC, along with other neurologic disorders [23, 28].

TSC is caused by a mutation in either the *TSC1* or the *TSC2* gene, the loss of which triggers constitutive activation of the mTOR signaling pathway, leading to abnormal cell growth/proliferation and the subsequent formation of hamartomatous lesions [25, 29]. The discovery of the relationship between *TSC1/TSC2* and mTOR has resulted in important clinical advances in the use of mTOR inhibitors, particularly sirolimus and its analog everolimus, for the treatment of several TSC manifestations. Sirolimus and everolimus both work by binding to and forming a complex with FK506-binding protein-12 (FKBP12) which then inhibits mTORC1 (Fig. 1) [24].

## mTOR inhibitors for the management of TSC-associated manifestations

### TSC-associated SEGA

Experience with sirolimus in treating SEGA was evaluated in case reports and as a secondary end point in a phase two trial with a small number of patients. In these cases, sirolimus demonstrated an observable regression of SEGA lesions [30–32].

Everolimus has been studied more extensively in treating SEGA through long-term phase 2 and 3 studies [33–36]. In a 6-month open-label phase 2 study consisting of 28 patients, everolimus demonstrated a significant reduction in tumor volume compared with baseline, with approximately 75% of patients experiencing a ≥ 30% reduction in SEGA volume and 32% experiencing a ≥ 50% reduction [33]; these reductions were sustained during the extension phase of the trial (median 5.65 years of treatment) [37]. In a randomized, double-blind, placebo-controlled, phase 3 study of 117 patients with SEGA associated with TSC, treatment with everolimus (median 9.6 months) was associated with a significantly higher SEGA response (≥ 50% reduction of SEGA volume) rate compared with placebo (35% vs. 0%; *p* < .0001) [35]. An analysis of 111 patients who received at least one dose of everolimus (in either the double-blind or a subsequent open-label phase) revealed that SEGA response increased to 57.7% over a median duration of 47.1 months (3.9 years), and the median reduction in SEGA volume was maintained, and even slightly increased, over the duration of the study [38]. Taken together, phase 2 and 3 clinical data on everolimus supported its use in the setting of TSC-associated SEGA, with the phase 2 results leading to the US Food and Drug Administration (FDA) approval of everolimus for the treatment of SEGA in pediatric and adult patients with TSC [39].

### TSC-associated renal angiomyolipoma

Everolimus was evaluated for the management of renal angiomyolipoma in the large phase 3 EXIST-2 trial and in a subset of the patients from the EXIST-1 trial who had SEGA and renal angiomyolipoma [40, 41]. In EXIST-2, the angiomyolipoma response rate (≥ 50% reduction in volume in absence of other factors) after approximately 8 months of treatment was 42% for patients taking everolimus compared with 0% in patients receiving placebo (*p* < .0001) [40], which increased to 54% in patients treated with everolimus for a median of 29 months [42], and 58% at the completion of the open-label extension phase (median exposure, 46.9 months) [43]. Based on the results from the core phase of EXIST-2, everolimus was approved by the FDA for the treatment of adult patients with renal angiomyolipoma and TSC [39]. Similar to the findings of EXIST-2, a subset of patients with SEGA and angiomyolipoma in EXIST-1 (largely pediatric population) reported angiomyolipoma response rates of 53.3% for everolimus and 0% for placebo after a median of 9.6 and 8.3 months of treatment, respectively; 80% of patients achieved a ≥ 50% reduction in renal angiomyolipoma volume after 48 weeks (11 months) of treatment [41].

Sirolimus has not been approved for the management of renal angiomyolipoma, but has been evaluated in several small open-label phase 2 clinical studies [32, 44–46]. Bissler et al. found that sirolimus reduced the size of angiomyolipoma lesions and improved lung function over 12 months of treatment [44]. However, 12 months after sirolimus was discontinued, lesion size and several lung function parameters approached baseline levels, suggesting that therapy with mTOR inhibition might necessitate long-term or indefinite use [44]. Davies et al. conducted a longer study and found that 50% of patients reported a positive angiomyolipoma response (disappearance of lesions or ≥ 30% reduction in sum of longest diameter of target lesions) over a 2-year period [45]. One phase 2, multicenter trial of sirolimus in adults with TSC evaluated the effects of sirolimus on multiple lesion types. Over a period of 1 year of treatment, they observed reductions in renal angiomyolipoma size, SEGA size, and liver angiomyolipoma size, with subjective improvement in skin lesions and reduction of vascular endothelial growth factor (VEGF) D [32].

### TSC-associated LAM

Sirolimus and everolimus have both been evaluated for the management of LAM in a number of studies consisting primarily of patients with sporadic LAM, although small numbers of patients with TSC-associated LAM were also included [47–50]. In the multicenter placebo-controlled MILES study, 89 patients with LAM (8 with a codiagnosis of TSC) receiving treatment with sirolimus (*n* = 46) over 12 months exhibited improvements in forced vital capacity (FVC) and quality of life, as well as stabilization of forced expiratory volume in 1 second (FEV_1_) [47]. These findings led to the FDA approval of sirolimus for the treatment of LAM [51]. Two retrospective studies also evaluated sirolimus in treating LAM [48, 49], reporting improved or stabilized lung function even at serum trough levels < 5 ng/mL [48], along with sustained effects over a treatment period of approximately 3.5 years [49].

Recently, everolimus was evaluated in a prospective study that included 24 patients (5 with TSC-LAM) and showed improvements in FEV_1_, stabilization of FVC, and reductions in VEGF-D and collagen IV; however, optimal dosing of everolimus for this indication needs further investigation [50]. As a result, everolimus has yet to receive approval for use in the LAM setting.

### TSC-associated seizures

Although no mTOR inhibitors are currently indicated specifically for the treatment of seizures associated with TSC, recent clinical evidence has shown promise for this use in this setting. The results of several small reports suggest that sirolimus may be effective for the treatment of TSC-associated seizures [52–54]. Sirolimus therapy given over 10 months in a 10-year-old girl reduced daily seizure activity from 5–10 times/day to 1–5 times/day and resulted in the cessation of seizure clusters [52]. A case series of seven children with TSC found that all patients experienced seizure control after 12 months of treatment with sirolimus [53]. A second case series of seven children with TSC and refractory seizures reported that most patients had 50% to 90% reductions in numbers of seizures [54]. In a recent, small, randomized trial of 23 children (ages 3 months to 12 years) with TSC, treatment with sirolimus decreased overall seizure frequency 41% over standard of care, but this change failed to reach statistical significance (*p* = .11) [55].

Use of everolimus in TSC-associated refractory seizures has also been evaluated [33, 56]. A prospective, phase 1/2 trial directly evaluating everolimus in managing refractory seizures associated with TSC showed a reduction in seizure frequency of ≥ 50% in 12 of 20 patients after 12 weeks of treatment [56]. In a phase 2 study, everolimus therapy was associated with a clinically relevant reduction in the overall frequency of clinical and subclinical seizures (median change, −1 seizure; *p* = .02) in patients with SEGA. Of the 16 patients for whom electroencephalographic data were available, seizure frequency decreased in nine patients after 6 months; five additional patients did not experience an event [33]. Results from the first phase 3 study to evaluate an mTOR inhibitor (everolimus) for refractory seizures associated with TSC were recently reported (ClinicalTrials.gov NCT01713946) [57]. This prospective, randomized, double-blind, multicenter study compared everolimus at two different trough levels (low exposure, 3–7 ng/mL; high exposure, 9–15 ng/mL) with placebo in reducing seizures (*N* = 366) when added to an existing antiepileptic drug regimen. After 18 weeks of treatment, the median percentage reduction in seizure frequency was significantly higher with everolimus (29.3% for everolimus low exposure and 39.6% for everolimus high exposure compared with 14.9% with placebo [*p* = .0028 and *p* < .0001, respectively]), and the proportion of responders (≥ 50% reduction in seizure frequency) was significantly greater with everolimus (28.2% for everolimus low exposure and 40% for everolimus high exposure compared with 15.1% with placebo [*p* = .0077 and *p* < .0001, respectively]) [57]. These preliminary findings indicate that adjunctive treatment with everolimus may be an effective option in reducing refractory seizures in patients with TSC.

### TSC-associated neuropsychiatric disorders (TAND)

mTOR inhibitors may also be a rational candidate for the management of neurodevelopmental/neuropsychiatric disabilities associated with TSC, including intellectual disability and autism. Indeed, a recent preclinical study of adult rats with *TSC2* mutations and developmental status epilepticus, and a case study of a patient with TSC both reported improvements in social deficit behaviors, including autism-related behaviors, following mTOR inhibitor therapy with everolimus [58, 59]. However, mTOR inhibitors have not been adequately evaluated or approved for the treatment of neurodevelopmental disabilities in TSC, especially in young infants. It is also essential that we establish the safety and overall impact of mTOR inhibitors in the pediatric population before larger, definitive clinical trials can be pursued. In the future, we await further information on effects of mTOR inhibitors on TSC-associated neuropsychiatric disorders, including secondary analyses from EXIST-3, and results from several phase 2 trials (NCT01289912, NCT01954693).

## Rationale for potential use of mTOR inhibitors in other novel indications

In addition to TSC, mTOR inhibition is being explored in other rare diseases for which mTOR dysregulation has been noted.

### Leigh syndrome

In Leigh syndrome, genetic defects result in disruption of mitochondrial function, which contributes to numerous health problems. Patients can have symptoms such as respiratory abnormalities, ocular and other cranial nerve palsies, involuntary movements, motor delays, intellectual disabilities, and seizures [60]. Although the time of onset can vary, it typically occurs in the first year of life. Leigh syndrome is characterized by diffuse multifocal spongiform degeneration in various parts of the brain, and many patients die within a few years after symptom onset [60].

In a preclinical study with *Ndufs4* knockout mice (the protein product of the *Ndufs4* gene is involved in the assembly, stability, and activity of complex I of the mitochondrial electron transport chain), rapamycin administration increased survivability and health [61]. The mechanism behind this is not entirely understood; however, it is believed that reduction of mTOR activity may shift cell metabolism toward amino acid catabolism and away from glycolysis and, thus, reduce the buildup of glycolytic intermediates that are associated with Leigh syndrome [61].

Additional research has suggested mTOR inhibition may aid in Leigh syndrome through preservation of adenosine triphosphate (ATP). Mitochondria provide energy to the cell through ATP, which has been found to be decreased in mitochondrial disorders; this leads to the degeneration of neurons, as in Leigh syndrome [62]. In an in vitro study, rapamycin was introduced to neuronal cells with mitochondrial defects, resulting in a significant rise in ATP level while protein production slowed [62]. It is theorized that the decrease in the energy-consuming process of protein synthesis with mTOR inhibition allows for more ATP to be spared [62].

Although investigation into the use of mTOR inhibitors in Leigh syndrome is at a very early stage, preclinical results are promising because there are currently no effective therapies for this disease.

### Down syndrome

Down syndrome is a genetic disorder associated with intellectual disability caused, in most cases, by trisomy of human chromosome 21 [63]. Down syndrome is characterized by abnormalities in dendritic morphology and synaptic plasticity, and mTOR is believed to be involved in the growth and branching of dendrites in the hippocampus [64]. The mTOR activity of dendrites in the hippocampus has been shown to be increased in a mouse model of Down syndrome [64]. This increase was subsequently reversed after administration of rapamycin. Studies are underway to investigate whether rapamycin can reverse learning deficits associated with Down syndrome.

Hyperactivation of the PI3K/Akt/mTOR pathway was also observed in autopsy samples of patients with Down syndrome compared with controls [65]. A causative factor of Down syndrome is hypothesized to be the triplication of amyloid-beta protein gene, resulting in excess protein proliferation. In combination with decreased autophagy as a result of increased mTOR activation, this may result in the accumulation of amyloid beta peptide in the brain and contribute to the neurodegenerative process and to eventual Alzheimer’s-like dementia in these patients [65]. Oxidative stress is also believed to have a role in neurodegenerative diseases such as Down syndrome. A mouse model of Down syndrome demonstrated that protein oxidation was increased possibly due to the decreased protective effect of autophagy as a result of mTOR pathway hyperactivation [66]. Signs of protein oxidation in cells were reduced when rapamycin was introduced [66].

### Neurofibromatosis types 1 and 2

Neurofibromatosis is an autosomal dominant genetic disorder that is further classified into subtypes 1 and 2. Neurofibromatosis types 1 and 2 are caused by inactivating mutations in *NF1* and *NF2* genes, respectively [67]. The loss of *NF1* encodes for the protein neurofibromin and results in development of neurofibromas on or around peripheral nerves, along with pigmented tumors of the skin and iris [67]. Plexiform neurofibroma occur in up to one-third of individuals with neurofibromatosis type 1 and can cause disfigurement, compression of other bodily structures, neurologic dysfunction, and pain [68]. Evidence suggests that neurofibromin is involved in negatively regulating the mTOR pathway. A phase 2 study involving patients with progressive plexiform neurofibromas treated with sirolimus showed a modestly increased time to progression [68]. However, a similar phase 2 study that evaluated sirolimus with nonprogressive plexiform neurofibromas showed that sirolimus did not cause any tumor shrinkage [69]. A case series involving patients with symptomatic plexiform neurofibromas showed that, although sirolimus did not shrink tumor volume, pain was alleviated [70]. The lack of an antitumor response with sirolimus in neurofibromatosis type 1 may be caused by alternative compensatory mechanisms (e.g., feedback activation of Akt activity) following mTOR inhibition [71].

Neurofibromatosis type 2, the rarer of the two subtypes, involves the loss of the *NF2* gene, which encodes for the regulator protein merlin. The loss of merlin leads to development of benign tumors called schwannomas, which can grow along auditory nerves, leading to hearing loss, and can compress nerves, leading to increased intracranial pressure, nerve dysfunction, and pain [67]. Similar to neurofibromin, merlin has been found to be a negative regulator of the mTOR pathway [72]. Rapamycin arrested schwannoma tumor growth in mice and in an in vitro model [72]. However, in a phase 2 study evaluating everolimus in the treatment of progressive vestibular schwannomas, none of the patients experienced a response (≥ 15% reduction in tumor volume) [73]. The activation of negative feedback loops following mTOR inhibition may also explain the limited efficacy of everolimus in vestibular schwannomas [73].

## Safety considerations with mTOR inhibition with TSC

Current research strongly suggests that mTOR inhibition, particularly with everolimus, seems to remain effective and safe over extended duration of treatment for certain TSC manifestations. However, adverse events (AEs) considered to be class effects of mTOR inhibitors should be taken into consideration when using sirolimus or everolimus, including noninfectious pneumonitis, infections, oral ulceration (e.g., stomatitis), impaired wound healing, and metabolic events (e.g., hyperglycemia, dyslipidemia) [39, 51]. These AEs may be appropriately managed by dose interruption or adjustment depending on severity of the event [39]. While long-term data on sirolimus in the TSC setting are lacking, clinical experience with everolimus in patients with TSC and renal angiomyolipoma or SEGA indicate that long-term everolimus therapy is generally well tolerated, with no new safety signals and the majority of AEs being mild to moderate in severity [36–38, 43, 74, 75].

## Long-term mTOR inhibition in TSC

Although mTOR inhibitors are being investigated for the previously mentioned novel indications simply as a possible treatment option at this stage, the use of mTOR inhibitors in TSC has already been established and necessitates further investigation into the effects of long-term treatment.

TSC is a lifelong condition that can manifest itself at a very early age, and it is possible that patients, including children, will need indefinite treatment with an mTOR inhibitor. Some of the later manifestations of TSC may be alleviated or prevented by early treatment with an mTOR inhibitor; however, initiation of therapy in children and young adolescents may have other consequences as well. Therefore, it is important to discuss the efficacy and safety of mTOR inhibitor treatment in youth and its potential long-term effects on growth and maturation.

If patients are to undergo mTOR treatment long-term, sustainability of response is important. The recent long-term data from EXIST-1 [38] and EXIST-2 [43] showed a sustained and more pronounced clinical benefit of everolimus compared with the shorter-term, primary analyses [35, 40]. Alternatively, in a subset of evaluable patients from EXIST-2 who were followed after discontinuation of everolimus (*n* = 7), angiomyolipoma lesion volume increased by more than 50% between everolimus discontinuation and 48 weeks after treatment [76]. These findings underscore the necessity of sustained treatment in TSC.

Although data on long-term exposure of mTOR inhibitors in youth, including effects on patient growth and sexual maturation, are limited, the EXIST-1 study reported that everolimus had no significant effect on puberty or development in patients with TSC after a median exposure of 47 months [38]. In addition, a retrospective analysis in a renal transplant population in which mTOR inhibitors were also used examined a cohort of 31 patients and reported that long-term mTOR therapy had no impact on growth and pubertal development after a mean follow-up of 4.9 years [77]. Further planned follow-up of the EXIST-1 population may provide additional evidence of the effect of everolimus on growth and sexual maturation in the youth population.

## Conclusions and future directions

Recent research in TSC has underlined the value of pursuing targeted therapies based on underlying molecular pathophysiology. TSC serves as a model for modification of acquired and inherited genetic defects causing brain dysfunction. A goal of current research is to identify the role of mTOR inhibition in neurologic and developmental disorders beyond that of TSC (e.g., Leigh syndrome, Down syndrome, and neurofibromatosis types 1 and 2), which nonetheless share a common feature of mTOR pathway hyperactivation. A better understanding of the molecular pathology of these seemingly diverse inherited and acquired brain diseases is necessary to achieve this goal. The recruitment of and performance of patients in basic science and particularly clinical studies is also a major challenge. However, available results in these areas show promise that, after further research, mTOR inhibition may eventually become a therapy option for these neurologic disorders where few options are currently available.

In addition to furthering research of mTOR inhibition in rare neurologic disorders, future research will also focus on defining the optimal use of mTOR inhibitors in TSC, including dosages for short- and long-term use, as well as age at which to initiate therapy. There has been longstanding interest in identifying treatment strategies for patients with TSC diagnosed at early ages where the potential effects of mTOR inhibitors or other therapies could drastically improve or even prevent the development of several TSC manifestations, including seizures, developmental delay, autism, renal disease, cutaneous tumors, and other lesions, by initiating treatment with mTOR inhibitors early in life. mTOR inhibitors are increasingly being used not only for the hamartomatous and oncologic manifestations of TSC, but also as adjunctive therapy for epilepsy and intellectual disability. Current research is expected to lead to a better definition of the roles of these therapies and their associated toxicities. As a result, the use of mTOR inhibition in TSC, including its use to prevent long-term morbidity, such as intellectual disability, autism, and refractory seizures, may be incorporated into clinical practice in the coming years.

## Abbreviations

AE: Adverse event
Akt: Protein kinase B
ATP: Adenosine triphosphate
FDA: Food and Drug Administration
FEV_1_: Forced expiratory volume in 1 s
FKBP12: FK506-binding protein-12
FVC: Forced vital capacity
IGF-1: Insulin-like growth factor-1
LAM: Lymphangioleiomyomatosis
mTOR: Mammalian target of rapamycin
PI3K: Phosphatidylinositol 3-kinase
RAPTOR: Regulatory associated protein of mTOR
Rheb: Ras homolog enriched in brain
RICTOR: Rapamycin-insensitive companion of mTOR
SEGA: Subependymal giant cell astrocytoma
SEN: Subependymal nodule
TAND: TSC-associated neuropsychiatric disorders
TSC: Tuberous sclerosis complex
VEGF: Vascular endothelial growth factor
